# Impairment of β-adrenergic regulation and exacerbation of pressure-induced heart failure in mice with mutations in phosphoregulatory sites in the cardiac Ca_V_1.2 calcium channel

**DOI:** 10.3389/fphys.2023.1049611

**Published:** 2023-02-08

**Authors:** Liam Hovey, Xiaoyun Guo, Yi Chen, Qinghang Liu, William A. Catterall

**Affiliations:** ^1^ Department of Pharmacology, School of Medicine, University of Washington, Seattle, WA, United States; ^2^ Medical Scientist Training Program, School of Medicine, University of Washington, Seattle, WA, United States; ^3^ Department of Physiology and Biophysics, School of Medicine, University of Washington, Seattle, WA, United States

**Keywords:** voltage-gated calcium channel, fight-or-flight, transverse aortic constriction, chronic heart failure, adrenergic, isoproterenol, protein phosphorylation, cAMP-dependent

## Abstract

The cardiac calcium channel Ca_V_1.2 conducts L-type calcium currents that initiate excitation-contraction coupling and serves as a crucial mediator of *β*-adrenergic regulation of the heart. We evaluated the inotropic response of mice with mutations in C-terminal phosphoregulatory sites under physiological levels of *β*-adrenergic stimulation *in vivo*, and we assessed the impact of combining mutations of C-terminal phosphoregulatory sites with chronic pressure-overload stress. Mice with Ser1700Ala (S1700A), Ser1700Ala/Thr1704Ala (STAA), and Ser1928Ala (S1928A) mutations had impaired baseline regulation of ventricular contractility and exhibited decreased inotropic response to low doses of *β*-adrenergic agonist. In contrast, treatment with supraphysiogical doses of agonist revealed substantial inotropic reserve that compensated for these deficits. Hypertrophy and heart failure in response to transverse aortic constriction (TAC) were exacerbated in S1700A, STAA, and S1928A mice whose *β*-adrenergic regulation of Ca_V_1.2 channels was blunted. These findings further elucidate the role of phosphorylation of Ca_V_1.2 at regulatory sites in the C-terminal domain for maintaining normal cardiac homeostasis, responding to physiological levels of *β*-adrenergic stimulation in the fight-or-flight response, and adapting to pressure-overload stress.

## 1 Introduction

The cardiac calcium channel Ca_V_1.2 conducts L-type calcium current, which initiates excitation-contraction coupling and constitutes a critical constituent of the cardiac β-adrenergic signaling cascade (Reuter 1983; Bers 2002; Weiss et al., 2013; Catterall, 2015). Phosphorylation by cAMP-dependent protein kinase (PKA) mediates the β-adrenergic increase in L-type calcium current (Tsien, 1973; McDonald et al., 1994). Truncation of the C-terminal domain (CT) of the pore-forming α1 subunit *in vitro* enhances Ca_V_1.2 channel activity in transfected cells (Wei et al., 1994). Up to 80% of the α1 subunit of Ca_V_1.2 in the heart is proteolytically processed near the center of the CT (De Jongh et al., 1996). The distal CT (dCT) binds to the proximal CT (pCT) and acts as a potent autoinhibitor of Ca_V_1.2 channel activity (Gao et al., 2001; Hulme et al., 2006a). Deletion of the dCT *in vivo* impairs Ca_V_1.2 expression in ventricular myocytes and blocks up-regulation of Ca_V_1.2 activity by β-adrenergic stimulation (Fu et al., 2011). These results implicate the CT in regulation of Ca_V_1.2 by β-adrenergic stimulation and PKA phosphorylation.

Regulation of the Ca_V_1.2 channel by PKA phosphorylation in transfected cells is impaired by mutation of the PKA consensus site at Ser1700 and the nearby casein kinase II (CKII) consensus site at Thr1704 (Fuller et al., 2010). Ventricular myocytes from mice with global alanine mutations Ser1700Ala (S1700A) and Ser1700Ala/Thr1704Ala (STAA) lose 2/3rds of their β-adrenergic-stimulated Ca_V_1.2 current (Fu et al., 2013; Fu et al., 2014). Importantly, this loss of β-adrenergic-stimulated Ca_V_1.2 current caused by the S1700A and STAA mutations takes place without significant effects on the expression or localization of Ca_V_1.2 in cardiac myocytes, confirming that the calcium conductance activity of the Ca_V_1.2 channels is reduced specifically by these mutations (Fu et al., 2013; Fu et al., 2014). Mice with S1700A and STAA mutations also have decreased fractional shortening of the ventricular wall, decreased ejection fraction, cardiac hypertrophy, and premature death at age 8–12 months (Yang et al., 2016). Although the findings of impaired regulation of Ca_V_1.2 current have been confirmed in a conditional knock-in STAA model, studies using Ca_V_1.2 α_1_-subunit with all predicted PKA phosphorylation sites and protease cleavage sites in the CT ablated reported preserved maximal β-adrenergic response (Yang et al., 2013; Katchman et al., 2017; Poomvanicha et al., 2017). These divergent results have generated active debate regarding the role of Ca_V_1.2 CT phosphorylation in β-adrenergic regulation in the heart (Catterall, 2015; Papa et al., 2022).

An additional PKA phosphorylation site on the Ca_V_1.2 CT at position Ser1928 is rapidly phosphorylated in cardiac myocytes during β-adrenergic regulation and has an important role in mediating Ca_V_1.2 upregulation by *β*-adrenergic stimulation in neurons and increased vascular reactivity in patients with diabetes (De Jongh et al., 1996; Hulme et al., 2006b; Patriarchi et al., 2016; Nystoriak et al., 2017). However, mice with global alanine substitution for Ser 1928 (S1928A) did not have substantial changes in baseline cardiac contractility, *in vivo β*-adrenergic response, or isoproterenol-induced enhancement of Ca_V_1.2 current in response to supramaximal doses of agonist in previous studies (Lemke et al., 2008).

In light of these discordant results from previous studies that were based primarily on *in vitro* measurements of Ca_V_1.2 currents, it is important to investigate the functional roles of these CT phosphoregulatory site *in vivo* in β-adrenergic regulation of cardiovascular homeostasis and cardiac contractility. In this study we evaluated the dose-dependence of the *in vivo* inotropic response to administration of *β*-adrenergic agonists in Ca_V_1.2/S1700A, STAA, and S1928A mice at physiological levels of stimulation, and we characterized the effects of heterozygous and homozygous S1700A and STAA mutations on cardiac function during chronic pressure overload. Our results support an important role for phosphorylation of CT sites in homeostatic regulation of Ca_V_1.2 activity, *β*-adrenergic regulation in response to physiological levels of stimulation *in vivo*, and resilience in the context of chronic pressure-overload stress.

## 2 Materials and methods

### 2.1 Animal models

C57BL/6 mice expressing Ala at positions 1700, 1700 and 1704, or 1928 in Ca_V_1.2 and their WT littermates were used in this study. Experimental procedures were approved by the Institutional Animal Care and Use Committee of University of Washington, and all studies were carried out in accordance with the approved guidelines. Mice were studied at age one to 4 months, well before signs of heart failure were evident at 6–9 months in S1700A and STAA mice.

### 2.2 Echocardiography

Echocardiography was performed with a VisualSonics Vevo 2100 imaging system under light isoflurane sedation (Li et al., 2016). Mice were anesthetized in the induction chamber with 2% isoflurane mixed with 1 L/min O2 by inhalation. Chest hair was removed by applying hair removal cream and skin was disinfected with 70% ethanol. Body temperature was maintained at 37°C *via* a heating pad and lamp. A nosecone to deliver 1.5% isoflurane mixed with 1 L/min O_2_ to maintain a steady level of anesthesia. Scanning was performed with a VisualSonics Vevo 2100 imaging system. M-mode ventricular dimensions were averaged from 3-5 cycles. Fractional shortening (FS) was calculated using left ventricular dimensions at the end of systole and diastole (LVES and LVED, respectively): FS = [(LVED - LVES)/LVED] x100 (%). Representative echocardiographic records are presented in Supplementary Figure S1.

### 2.3 Transverse aortic constriction

Transverse aortic constriction was performed under anesthesia by 1.5% isoflurane mixed with 1 L/min O_2_. The transverse aortic arch was visualized through a median sternotomy, and 7–0 silk ligature was tied around the aorta (constriction made by 27-gauge needle) between the right brachiocephalic and left common carotid arteries (Li et al., 2016). Pressure gradients (mm Hg) were calculated from the peak blood velocity (Vmax) (m/s) measured by Doppler across the aortic constriction using the modified Bernoulli’s equation: (PG = 4 x V_max_^2^), which was equivalent in all groups of TAC stimulated mice.

### 2.4 Heart weight

Mice were weighed, euthanized, and the hearts were isolated and rinsed in PBS to remove blood. The hearts were blotted dry, separated into ventricle and atrium, and weighed. Ventricular weight was normalized to bodyweight.

### 2.5 β-Agonist administration

A racemic mixture of isoproterenol (Sigma-Aldrich #I6504) dissolved in 0.9% saline was used. After baseline echocardiographic assessment, S1700A, STAA, or S1928A mice aged 5–15 weeks were given 0.25–100 μg/kg isoproterenol *via* intraperitoneal injection. Post-injection echocardiographic measurements were made 2 minutes after drug administration.

### 2.6 Quantification of cardiac hypertrophic markers

Ventricular tissue was isolated from mice 4 weeks after TAC surgery and from littermates that did not receive surgery. Total cDNA libraries were generated using RNA isolation and reverse transcription. Quantitative PCR was performed using primers for ANP (fwd: 5′-TCG​TCT​TGG​CCT​TTT​GGC​T-3′, rev: 5′-TCC AGG TGG TCT AGC AGG TTC T-3′), BNP (fwd: 5′- AAG​TCC​TAG​CCA​GTC​TCC​AGA-3, rev: 5′- GAG CTG TCT CTG GGC CAT TTC -3′), β-MHC (fwd: 5′-ATG​TGC​CGG​ACC​TTG​GAA​G-3′, rev: 5′-CCT CGG GTT AGC TGA GAG ATC A-3′), and GAPDH (fwd: 5′CAT GGC CTT CCG TGT TCC TA 3′, rev: 5′CCT GCT TCA CCA CCT TCT TGA T 3′). Normalized amplification threshold (ΔC_T_) values were measured for each sample relative to GAPDH. ΔΔC_T_ was calculated as the difference in ΔC_T_ between post-TAC and non-TAC animals, and expression fold change (R_Q_) was calculated *via* logarithmic transformation 2^−ΔΔCT^.

### 2.7 Statistical analysis

Statistical significance was determined *via* Analysis of Variance (ANOVA) of all genotypes for a given condition, with pairwise differences determined with Tukey HSD post-hoc tests unless otherwise noted. Results are presented as mean ± standard errors.

## 3 Results

### 3.1 Baseline defects in homozygous and heterozygous Ca_V_1.2 phosphomutant mice

**FIGURE 1:**
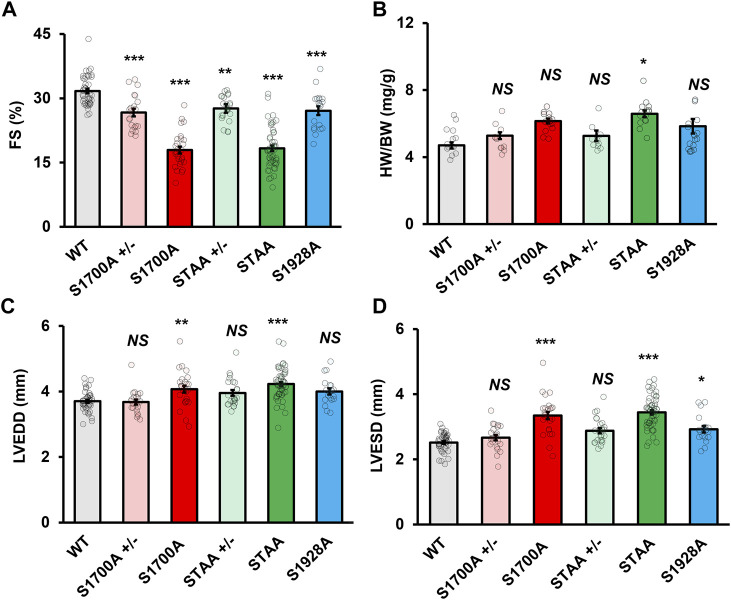
Impaired baseline systolic function in mice with heterozygous and homozygous Ca_V_1.2 phosphoregulatory site mutations. Left-ventricular **(A)** fractional shortening, **(B)** heart weight, **(C)** end-diastolic diameter and **(D)** end-systolic diameter in young WT, heterozygous and homozygous S1700A and STAA mice, and homozygous S1928A mice. Statistical significance determined *via* ANOVA and post-hoc Tukey HSD comparison to WT. Error bars are S.E.M.; *N* = 41 (WT), 28 (S1700A), 53 (STAA), 19 (S1928A), 23 (STAA +/−), 21 (S1700A +/−); *p < 0.05, **p < 0.01, ***p < 0.001.

Previous cellular physiology studies showed that the Ser1700A and STAA phosphoregulatory mutations reduced basal Ca_V_1.2 currents in cardiomyocytes (Fu et al., 2013; Fu et al., 2014). Based on those results, we investigated the effects of the Ser1700A and STAA mutations on baseline regulation of cardiac contractility. Ca_V_1.2 mutant mice with global alanine substitutions at positions Ser1700, Ser1700/Thr1704, and Ser1928 were produced as described (Lemke et al., 2008; Fu et al., 2013; Fu et al., 2014). Baseline functional and morphologic characteristics were assessed in male and female mice aged one to 4 months using echocardiography under light isoflurane sedation (Supplementary Tables S1, S2). Homozygous S1700A and STAA mice showed lower baseline left-ventricular fractional shortening (FS) (Figure 1A, 17.9% ± 0.8% and 18.3% ± 0.6%, respectively), compared with WT animals (31.3% ± 0.6%, *p* < 0.001). Similarly, heterozygous S1700A and STAA mice had significantly decreased FS (Figure 1A: 26.7% ± 0.9%, *p* = 0.001; and 27.6% ± 0.6%, *p* = 0.016, respectively). Moreover, homozygous S1928A mice also had consistently reduced FS (Figure 1A: 27% ± 1%, *p* = 0.003). Homozygous STAA mice had significantly increased heart weight/body weight ratio (HW/BW), while homozygous S1700A mice showed a nearly-significant trend toward hypertrophy (Figure 1B). The homozygous S1700A and STAA mice also showed clear evidence of ventricular dilation at baseline, with increased left-ventricular end-diastolic diameter (LVEDD) (Figure 1C: 4.2 ± 0.1 mm, *p* = 0.006; and 4.22 ± 0.06 mm, *p* < 0.001, respectively) and increased left ventricular end-systolic diameter (LVESD) (Figure 1C: 3.3 ± 0.1 mm, *p* < 0.001; and 3.44 ± 0.07 mm, *p* < 0.001, respectively) compared with WT (LVEDD: 3.70 ± 0.05 mm; LVESD: 2.52 ± 0.04 mm). Interestingly, while heterozygous S1700A (S1700A^+/−^), STAA (STAA^+/−^) and homozygous S1928A mice did not have hypertrophy or significantly elevated LVEDD, S1928A mice did have increased LVESD (Figure 1D: 2.9 ± 0.1 mm, *p* = 0.033), compared with WT, as also observed for homozygous S1700A and STAA mice. Other signs of generalized hypertrophy of the left ventricle, such as increased width of the posterior wall or interventricular septum, were not evident in the mutant animals at baseline (Supplementary Table S3). There also were no notable differences between male and female mice in the echocardiographic measurements, with the exception of the FS in the S1928A mice, which was only statistically significant when comparing female S1928A and WT mice (Supplementary Table S4).

Heart weight normalized to bodyweight was significantly higher in mice with homozygous S1700A and STAA mutations (Supplementary Table S1; 6.2 ± 0.1 mg/g bodyweight, *p* < 0.001; and 6.6 ± 0.2 mg/g, *p* < 0.001, respectively), compared with WT animals (4.7 ± 0.2 mg/g). The homozygous S1700A and STAA mice also appeared to have increased left ventricular end-systolic diameter (LVESD) and HW/BW, although the heart weight elevation was only statistically significant in STAA mice. Our results show that baseline contractility was significantly impaired in all five CT phosphomutant mouse lines, suggesting that phosphorylation of the Ca_V_1.2 CT sites is required for normal cardiac homeostasis.

### 3.2 *In Vivo* chronotropic response to β-adrenergic stimulation

The baseline heart rate was significantly elevated in the S1700A and STAA mice (Supplementary Table S5; Supplementary Figure S2A, red, green; 515 ± 6 bpm, *p* < 0.001; and 504 ± 7 bpm, *p* < 0.001) compared with WT (466 ± 7 bpm), but not in heterozygous animals. Although administration of low doses (0.25 μg/kg) and high doses (100 μg/kg) of isoproterenol had a similar impact on heart rate in WT and mutant animals, the intermediate dose (1 μg/kg) resulted in significantly higher heart rates in S1700A and STAA mice (Supplementary Table S5; Supplementary Figure S2; 6.3 ± 0.3 × 10^−2^ bpm, *p* = 0.022; and 6.3 ± 0.2 × 10^−2^ bpm, *p* = 0.011) compared with WT (5.3 ± 0.2 × 10^−2^ bpm). Importantly, the chronotropic response to treatment with isoproterenol was similar to WT or greater than WT for S1700A, STAA, and STAA^+/−^ mice (Supplementary Figure S2; Supplementary Table S5). The normal *β*-adrenergic response for heart rate in WT and mutant mice indicates that the function of the *β*-adrenergic receptor and its downstream signaling to G proteins, cAMP, and the hyperpolarization and cyclic nucleotide-regulated (HCN) channels is also normal in S1700A and STAA mice.

Homozygous S1928A animals had depressed heart rate compared with WT animals (410 ± 8 bpm, *p* < 0.001) at baseline and after administration of 0.25 μg/kg of isoproterenol (Supplementary Table S5; Supplementary Figure S2, light blue; S1928A: 4.4 ± 0.2 × 10^−2^ BPM; WT: 5.2 ± 0.2 × 10^−2^ BPM; *p* = 0.040). These results suggest a distinct mechanism of response compared to the S1700A and STAA mice.

### 3.3 *In Vivo* inotropic response to β-adrenergic stimulation

Previous studies primarily describe the effects of administration of *supraphysiologic* doses of *β*-adrenergic agonists to mice with mutations in Ca_V_1.2 phosphoregulatory sites, often exceeding 100μM g of drug per kg body weight (Lemke et al., 2008; Poomvanicha et al., 2017). During the *in vivo* fight-or-flight response of mice to male-male fighting or enduring restraint stress, circulating plasma levels of epinephrine are in the range of 60–180 ng/kg (Hucklebridge et al., 1973; Kvetnansky et al., 2006). Administration of the lowest dose of the racemic mixture of isoproterenol used in this study would achieve a plasma concentration as high as 125 ng/kg of active isomer, whereas previous studies of Ca_V_1.2 mutant mice used doses that would achieve 100-fold higher circulating concentrations. Synaptically-released norepinephrine also contributes significantly to the cardiac fight-or-flight response in rodents, as demonstrated in studies of β-adrenergic stimulation of adrenalectomized rats (Phornchirasilp and Matangkasombut, 1982). As isoproterenol is a more potent β_1_-receptor agonist, the circulating concentration of 125 ng/kg achieved in our experiments would likely be sufficient to mimic the combined contributions of circulating epinephrine plus synaptically released norepinephrine in the cardiac stress response *in vivo*.

**FIGURE 2:**
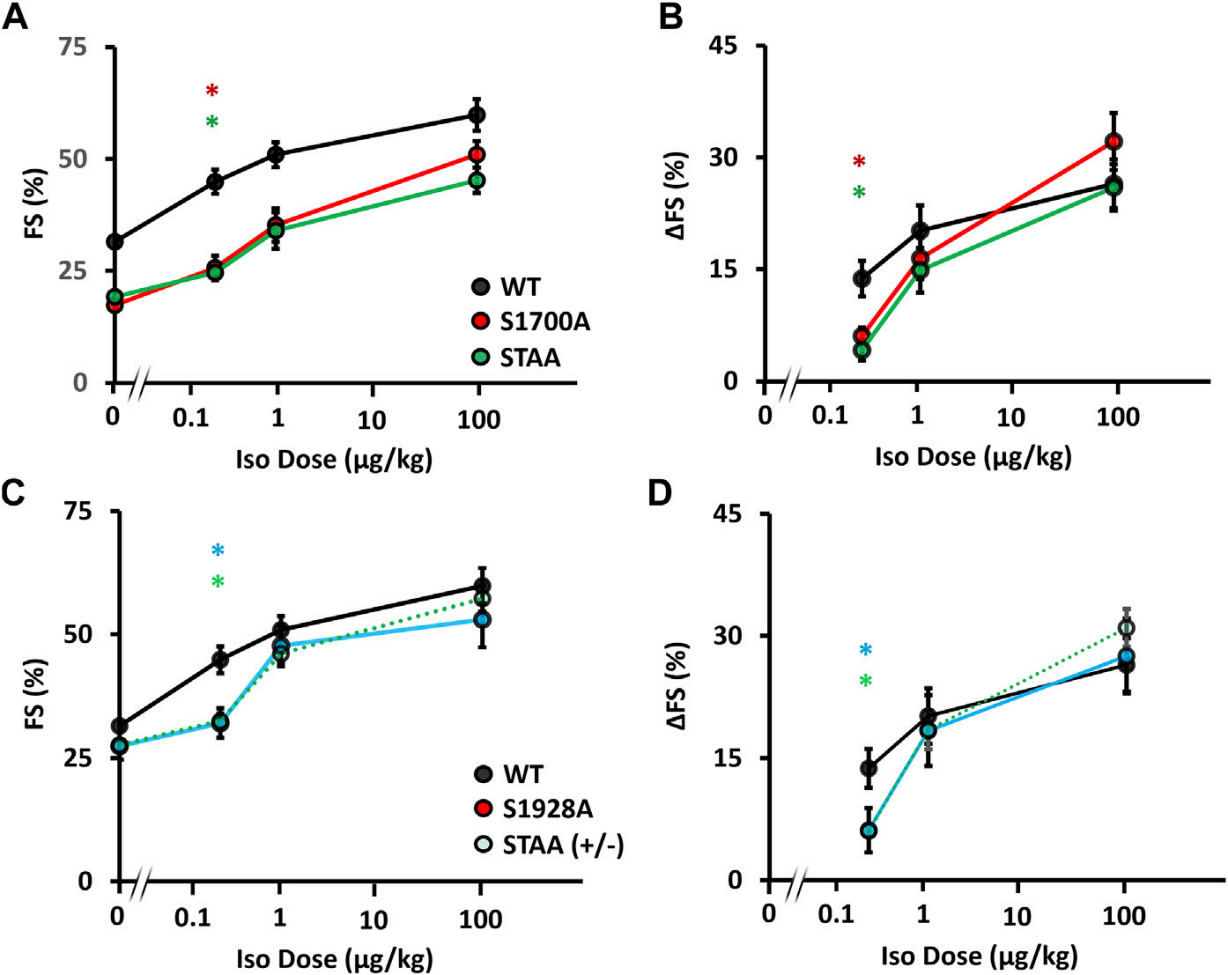
Inotropic dose-response to β-adrenergic stimulation in mice with Ca_V_1.2 phosphoregulatory site mutations. Isoproterenol dose-response assessed *via* **(A)** fractional shortening, and **(B)** change in fractional shortening in WT, S1700A, STAA mice, and **(C)** fractional shortening and **(D)** change in fractional shortening in WT, STAA +/−, and S1928A mice, in response to intraperitoneal drug administration. Statistical significance determined *via* ANOVA with Tukey post-hoc tests. Error bars are S.E.M.; N_0.25 μg/kg_ = 11, 8, 6, 8, 8 (for WT, S1700A, STAA, S1928A, STAA +/−, respectively); N_1 μg/kg_ = 6, 7, 8, 6, 9 (for WT, S1700A, STAA, S1928A, STAA +/−, respectively); N_100 μg/kg_ = 7, 7, 7, 7, 11 (for WT, S1700A, STAA, S1928A, STAA +/−, respectively); *p < 0.05 (red *S1700A vs. WT, light-green STAA ± vs. WT, dark green STAA vs. WT, light-blue S1928A vs. WT).

To investigate the impact of phosphoregulatory Ca_V_1.2 mutations on cardiac adrenergic regulation at physiological levels of *β*-adrenergic stimulation, we compared the *in vivo* inotropic responses of mice at two physiologic doses (0.25 μMg/kg and 1 μMg/kg) and one supraphysiologic dose (100 μMg/kg) of isoproterenol (Figures 2A, B). In the S1700A and STAA mice, baseline FS in the absence of isoproterenol was significantly lower than controls (WT, 31.3% ± 0.6%; STAA, 17.9% ± 0.8%; S1700A 18.3% ± 0.6%; Figure 2A). Treatment with 0.25 μMg/kg isoproterenol produced a smaller increment in FS for STAA and S1700A than observed in the WT mice (Figure 2A). This difference is more evident in Figure 2B where the increment in FS caused by isoproterenol (ΔFS) in these mutants is plotted vs. isoproterenol dose (6% ± 1%, *p* = 0.048 and 4% ± 1%, *p* = 0.018 respectively) and compared with WT (14% ± 3%). The stimulated FS values in these mice were still significantly depressed compared with stimulated WT animals at the intermediate 1 μMg/kg dose (Figure 2A: S1700A, 26% ± 3%, *p* < 0.001; STAA, 25% ± 1%, *p* < 0.001; WT, 45% ± 3%). However, the increase in ΔFS caused by treatment with 1 μMg/kg isoproterenol was smaller and more variable in S1700A and STAA mice and did not reach statistical significance (WT: 20 ± 3; S1700A: 16 ± 3, *p* = 0.93; STAA: 15 ± 3, *p* = 0.83). On the other hand, administration of the highest dose of isoproterenol (100 μMg/kg), consistently produced larger increases in FS (Figure 2A) and ΔFS (Figure 2B: S1700A, 32% ± 4%; *p* = 0.79; STAA, 26% ± 3%; *p* = 1.0) in mutant mice, which were comparable to WT (26% ± 3%). These results show that the *β*-adrenergic response is impaired by these phosphoregulatory site mutations in Ca_V_1.2 at low physiological doses of isoproterenol, whereas higher doses are able to overcome the deficit in *β*-adrenergic signaling caused by the mutations.

**FIGURE 3:**
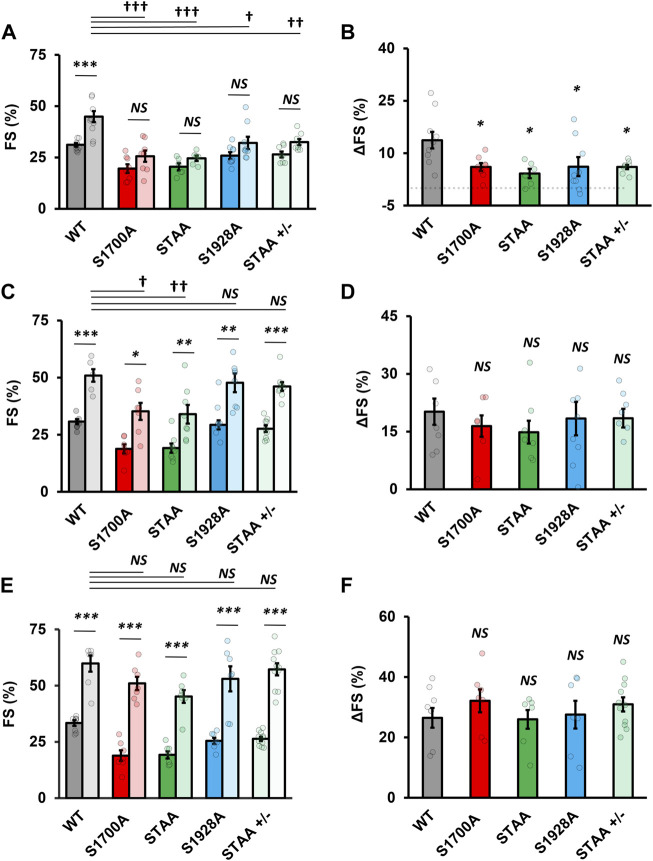
Impaired inotropic response to physiologic range β-adrenergic stimulation in mice with Ca_V_1.2 phosphoregulatory site mutations. **(A)** Fractional shortening and **(B)** change in fractional shortening in WT, S1700A, STAA, and S1928A, and STAA heterozygous (+/−) mice before and after 0.25 μg/kg intraperitoneal isoproterenol administration. **(C)** Fractional shortening and **(D)** change in fractional shortening intraperitoneal before and after 1 μg/kg drug administration. **(E)** Fractional shortening and **(F)** change in fractional shortening intraperitoneal before and after 100 μg/kg drug administration. Statistical significance determined *via* ANOVA with Tukey post-hoc tests. Error bars are S.E.M.; N_0.25 μg/kg_ = 11, 8, 6, 8, 8 (for WT, S1700A, STAA, S1928A, STAA +/−, respectively); N_1 μg/kg_ = 6, 7, 8, 6, 9 (for WT, S1700A, STAA, S1928A, STAA +/−, respectively); N_100 μg/kg_ = 7, 7, 7, 7, 11 (for WT, S1700A, STAA, S1928A, STAA +/−, respectively). *p* < 0.05, **p < 0.01, ***p < 0.001 for comparisons against baseline, ^†^ *p* < 0.05, ^††^ *p* < 0.01, ^†††^ *p* < 0.001 for comparisons against stimulated WT.

Longstanding impairment in contractility can lead to *β*-adrenergic desensitization through mechanisms that are unrelated to Ca_V_1.2 phosphoregulation. Therefore, we also evaluated the impact of heterozygous STAA mutation and homozygous S1928A mutation on the β-adrenergic inotropic response because these mice have robust contractile function at baseline and do not develop CHF. We again observed a deficit in the contractile response to low-dose isoproterenol stimulation (Figures 2C, D; Figures 3A, B: stimulated FS, S1928A, 34% ± 2%, *p* = 0.015; STAA^+/−^, 33% ± 2%, *p* = 0.008) compared with WT (45% ± 3%). Unlike S1700A and STAA homozygous animals, S1928A and STAA^+/−^ mice responded normally to the intermediate dose and high doses of isoproterenol, achieving FS comparable to WT (Figures 3C, D). These results show that mice with the STAA mutation in one allele or the homozygus S1928A mutation also have a measurable deficit in *β*-adrenergic regulation at the lowest dose of agonist.

As both the chronotropic and inotropic responses to *β*-adrenergic stimulation use the *β*-receptor>Gs>adenylate cyclase>cAMP signaling pathway, our results showing normal *β*-adrenergic regulation of beating rate provide strong evidence that this signaling pathway is intact in the heart. Therefore, these results support the conclusion that the effects of mutations of Ser1700 and Thr1704 are caused by altered PKA regulation of Ca_V_1.2 function rather than by defects in other components of the *β*-adrenergic signaling cascade. Nevertheless, a significant caveat remains, because it is conceivable that upstream signaling changes in the pathway between the *β*-adrenergic receptor and Ca_V_1.2 channels might influence regulation of contraction in the ventricular myocyte but not regulation of heart rate in the sinoatrial node.

### 3.4 Exacerbated heart failure in S1700A and STAA mice after aortic constriction

**FIGURE 4:**
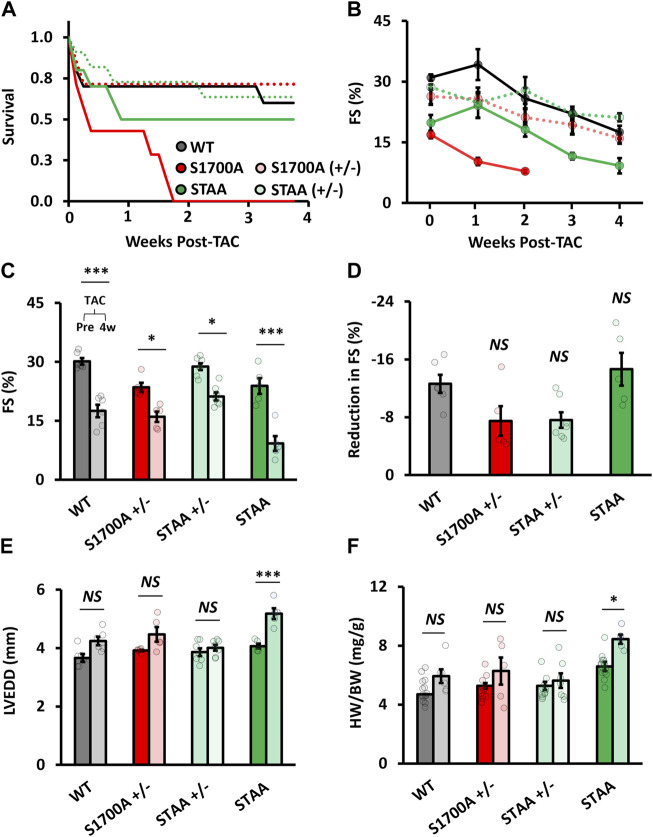
Hypertrophy and ventricular dilation after TAC in mice with homozygous mutations of Ca_V_1.2 phosphoregulatory sites. **(A)** Survival rates after TAC for WT (gray markers), heterozygous SA (light red) and STAA (light green), and homozygous SA (red) and STAA (green) mice aged 50–90 days. Left-ventricular **(B)** fractional shortening measured at 1 week intervals following TAC surgery. **(C)** Fractional shortening, **(D)** reduction in fractional shortening, **(E)** left-ventricular end-diastolic diameter, and **(F)** heart-weight 4 weeks after TAC surgery (right bars) and in non-TAC control animals (left bars) in WT and homozygous STAA mice. Error bars are S.E.M.; N_4 Weeks_ = 6 (WT), 5 (STAA), 7 (STAA^+/−^), 5 (S1700A^+/−^). *p < 0.05, **p < 0.01, ***p < 0.001.

Our previous results showed that S1700A and STAA mice develop severe hypertrophy and lethal CHF after 100 days of age (Yang et al., 2016). However, these mice are overtly normal at younger ages. Because *β*-adrenergic antagonists are beneficial in established CHF, we tested whether reduced β-adrenergic regulation caused by mutation of phoshoregulatory sites on Ca_V_1.2 channels would be beneficial in pressure overload-induced CHF or would exacerbate this pathological condition. After baseline echocardiography, heterozygous and homozygous S1700A and STAA mice aged 50–90 days underwent transverse aortic constriction (TAC) surgery to induce persistent cardiac pressure-overload stress. Cardiac function and morphology were assessed weekly and animals were euthanized 4 weeks after surgery (Figure 4).

S1700A and STAA mice had lower survival rates than WT following TAC (Figure 4A, red and green). Surprisingly, homozygous mice with only the S1700A mutation experienced significantly lower postsurgical survival compared with STAA mice, with no surviving animals by 4 weeks post-surgery (Figure 4A). Due to the high mortality in the S1700A mice, we discontinued further surgeries on this mouse line. In contrast, 50% of the STAA animals that underwent surgery survived until 4 weeks, compared with 60%–70% of the WT and heterozygous mice (Figures 4A, C).

Four weeks after surgery, surviving STAA mice had markedly decreased fractional shortening (Figure 4B; 5A: 9% ± 2%, *p*=<0.001) compared with WT animals (18% ± 2%), although there was a comparable absolute loss of contractility measured as reduction in FS (Figure 4D: reduction in FS, STAA, −15 ± 2; WT,-1 ± 1, *p* = 0.82). However, STAA mice experienced exacerbated cardiac remodeling compared with controls. Indeed, we observed a significant increase in ventricular dilation in STAA mice (Figures 4E, F: LVEDD, STAA pre-TAC, 4.07 ± 0.09 mm vs. post-TAC, 5.2 ± 0.1; *p* < 0.001), whereas WT mice experienced a moderate increase in ventricular size (Figure 4E: WT, pre-TAC, 3.7 ± 0.1 mm vs. post-TAC, 4.2 ± 0.1, *p* = 0.081). STAA mice also had increased HW/BW compared with WT mice (Figure 4F: HW/BW; STAA, no TAC, 6.6 ± 0.2 mg/g vs. post-TAC, 8.4 ± 0.3; *p* < 0.047; WT no-TAC 4.7 ± 0.2 mg/g vs. post-TAC: 5.9 ± 0.5; *p* = 0.42). Evidently, mutation of the CT phosphoregulatory sites substantially exacerbates the adverse effects of TAC, revealing a significant loss of cardiovascular resilience.

Expression of hypertrophic markers was evaluated in both WT and STAA mice post-TAC. Compared with age-matched control animals without TAC, we observed greater relative quantification (R_Q_) values of atrial natriuretic peptide (ANP) mRNA in the STAA animals (R_Q_ ANP: STAA, 7.74 ± 1.94 vs. WT, 2.72 ± 0.41; *p* = 0.039). BNP and β-MHC both showed a trend toward elevation in WT and STAA post-TAC animals that did not reach significance.

Unlike homozygous S1700A and STAA animals, young heterozygous S1700A and STAA mice tolerated TAC well, and experienced comparable changes in FS, HW/BW, and LVEDD as observed for WT animals (Figure 4). In fact, the STAA^+/−^ cohort exhibited a trend towards a smaller loss of contractility compared with WT animals (Figure 4D: ΔFS, STAA^+/−^, −7 ± 1%, *p* = 0.12; S1700A^+/−^, −7 ± 2%, *p* = 0.16; WT, −13% ± 1%). Together, these results support the conclusion that the STAA mutation exacerbates hypertrophy following TAC.

## 4 Discussion

### 4.1 Mutations of CT phosphoregulatory sites impair baseline cardiovascular performance

Mice with homozygous SA and STAA mutations have substantially impaired cardiac performance, and develop pathological hypertrophy and heart failure as they age (Fu et al., 2013; Fu et al., 2014; Yang et al., 2016), which is consistent with later work using a different STAA mouse line (Poomvanicha et al., 2017). In the *in vivo* studies presented here, we found that mice with homozygous S1700A and STAA mutations have significantly impaired baseline cardiovascular performance, as revealed by reduced FS and increased LVESD and LVEDD. Mice with heterozygous S1700A and STAA mutations had less marked depression of contractile function, which is not sufficient to cause significant hypertrophy or ventricular dilation. In contrast to earlier work (Lemke et al., 2008), we found that Ca_V_1.2/S1928A mice also had a significant reduction in baseline contractility, although not sufficient to cause progressive dysfunction and heart failure. Thus, mice with long-term heterozygous loss of phosphorylation of Ser1700, Thr1704, and homozygous loss of Ser1928 phosphorylation are able to compensate and retain a normal range of cardiac function, whereas the homozygous mutations S1700A and STAA lead to severe systolic dysfunction, hypertrophy, and premature death.

### 4.2 CT phosphoregulatory mutations lead to compensatory up-Regulation of contractility

Previous studies of S1700A and STAA mice revealed compensatory up-regulation of PKA phosphorylation of phospholamban, ryanodine RyR2 receptors, and troponin-I as well as increased calcium levels in the sarcoplasmic reticulum. These compensatory effects would all increase the contractile response to *β*-adrenergic stimulation and thereby reduce the negative effects of the S1700A and STAA mutations on contractile performance. Consistent with this mechanism, the inhibitory effects of the S1700A and T1704 phosphoregulatory mutations on contractility of dissociated cardiomyocytes are significantly blunted compared to their inhibitory effects on Ca_V_1.2 currents in the same cells (Fu et al., 2013; 2014; Yang et al., 2016). These observed compensatory effects on cellular contractility would reduce the impact of CT phosphoregulatory mutations on the inotropic response to *β*-adrenergic stimulation *in vivo*, leading to underestimation of the effects of these mutations *per se* on ventricular performance.

### 4.3 Mutations of CT phosphoregulatory sites impair physiological β-adrenergic response

*In vivo* studies of *β*-adrenergic regulation of cardiovascular function in mice with Ca_V_1.2 mutations have typically been performed at very high doses of isoproterenol (≥100 μMg/kg bodyweight), well beyond the estimated range of *β*-adrenergic stimulation *in vivo* (Lemke et al., 2008; Poomvanicha et al., 2017). In contrast, we investigated the response of homozygous S1700A, STAA, and S1928A mice at a range of doses of isoproterenol (0.25–100 μg/kg bodyweight) that mimic the range of physiological stimulation *in vivo*. Notably, we found that the lowest dose of isoproterenol (0.25 μg/kg) elicited less than half the contractile response measured as ΔFS compared to WT mice. Our results indicate that the contribution of phosphorylation of Ser1928 to the cardiac adrenergic response has been underestimated previously due to the use of supraphysiological stimulation by high doses of *β*-adrenergic agonist. Altogether, our findings show that phosphorylation of Ser1700, Ser1704, and Ser1928 all contribute significantly to the fight-or-flight response in the context of physiological levels of *β*-adrenergic stimulation. It is likely that the effects of phosphorylation of these sites *per se* is underestimated in our echocardiographic experiments because of the physiological compensation noted above.

### 4.4 Supraphysiological *β*-adrenergic stimulation overcomes the effects of CT mutations

At high (100 μg/kg) doses of isoproterenol, all four genotypes (WT, S1700A, STAA, S1928A) reached maximal ejection fractions of >90%. This preserved *β*-adrenergic reserve may be the result of long-term compensatory processes, as earlier work has shown that the effects of a shorter-term conditional knock-in mutation of the STAA sites in a different genetic background does not have a robust *β*-adrenergic response (Poomvanicha et al., 2017). Evidently, phosphorylation of these CT sites is required for normal response to physiological levels of *β*-adrenergic stimulation, but that deficit can be overcome by treatment with supramaximal doses of *β*-adrenergic agonist.

Studies in other experimental systems have not found similar importance of phosphorylation of Ser1700, Thr1704, and Ser1928 in regulation of cardiac Ca_V_1.2 channels by the *β*-adrenergic signaling pathway (Lemke et al., 2008; Yang et al., 2013; Katchman et al., 2017). In studies of a similar S1928A mouse model and a different STAA mouse model, it was reported that the *β*-adrenergic regulation of Ca_V_1.2 in dissociated cardiac myocytes and *in vivo* remained intact; however, only high concentrations/doses of *β*-adrenergic agonists were tested (0.2–1 μM isoproterenol *in vitro*; 100–500 μMg/kg isoproterenol *in vivo*) (Lemke et al., 2008; Qian et al., 2017). As we find that supraphysiological stimulation by high doses of isoproterenol can overcome the deficit caused by mutation of the CT phosphoregulatory sites, it is important to re-examine these previous results with physiologically relevant levels of *β*-adrenergic stimulation. A different experimental approach also found no evidence for *β*-adrenergic regulation by phosphorylation of these CT regulatory sites (Yang et al., 2013; Katchman et al., 2017). In those studies, 92% of endogenous Ca_V_1.2 current was blocked with a dihydropyridine antagonist, permitting studies of transgenic, dihydropyridine-insensitive Ca_V_1.2 channels with phoshoregulatory site mutations in dissociated cardiac myocytes with endogenous WT Ca_V_1.2 channels. Unfortunately, these studies also used a high concentration of isoproterenol (0.2 μM), far above the level of catecholamine observed *in vivo* during physiological regulation, and the remaining 8% of uninhibited endogenous WT Ca_V_1.2 channels may have contributed to the *β*-adrenergic regulation observed in the mutant mice at supraphysiological levels of stimulation.

### 4.5 Phosphorylation of CT regulatory sites contributes to cardiovascular resilience

Mice whose cardiac function is stressed by TAC are adversely affected by mutations that prevent phosphorylation of CT phosphoregulatory sites. Mice with homozygous S1700A mutations had markedly lower survival rate 4 weeks following TAC compared with WT and heterozygous animals. Mice with homozygous STAA mutations had lower survival rate and increased impairment of ventricular function. Heterozygous mutations of these sites had milder effects. These findings show that phosphorylation of the CT phosphoregulatory sites of Ca_V_1.2 contributes substantially to resilience to cardiovascular stress and provide additional evidence for the tolerability of partial, but not total, loss of PKA phosphorylation at Ser1700 and Thr1704.

### 4.6 Convergent regulation by direct PKA and indirect rad phosphorylation pathways

Recent reports indicate that phosphorylation of the small GTPase Rad (Ras-associated with diabetes) plays an important role in regulation of Ca_V_1.2 channels (Liu et al., 2020; Katz et al., 2021; Papa et al., 2021; Papa et al., 2022). One study of Ca_V_1.2 channels expressed in a mammalian noncardiac cell line reported no regulation of Ca_V_1.2 by activation of PKA in the absence of Rad, and another study of Ca_V_1.2 channels expressed in *Xenopus* oocytes estimated that 80% of Ca_V_1.2 upregulation in response to *β*-adrenergic stimulation was Rad-dependent and 20% was Rad-independent. However, these studies of Rad regulation used overexpressed full-length Ca_V_1.2 as a regulatory target, whereas cardiac Ca_V_1.2 is proteolytically processed *in vivo*, and the more abundant form in the heart is the truncated Ca_V_1.2Δ1800 in complex with the proteolyzed, auto-inhibitory distal C-terminal domain (De Jongh et al., 1996; Hulme et al., 2006a; Ahern et al., 2019). Recent studies of this isoform of Ca_V_1.2 in transfected human cells revealed convergent regulation by PKA phosphorylation of the Ca_V_1.2 CT and by PKA phosphorylation of Rad, indicating that these two independent signaling pathways have synergistic roles in modulating Ca_V_1.2 channels (Hovey et al., 2022).

## 5 Conclusion

We demonstrate definitively in a well-validated mouse genetic model that phosphorylation of the regulatory sites in the Ca_V_1.2 CT is indeed required for normal basal function and *β*-adrenergic regulation in response to physiological levels of stimulation *in vivo*, as implied by the hypertrophy and lethal heart failure caused by these mutations. Phosphorylation of these regulatory sites is also important in sustaining resilience during chronic pressure-overload stress. In contrast, an *in vivo* model of cardiac-specific Rad knockout mice has preserved *β*-adrenergic regulation of calcium transients and contractile responses, suggesting a more limited role for the Rad pathway in *β*-adrenergic regulation of cardiovascular homeostasis *in vivo* in these mice (Ahern et al., 2019). These results from *in vivo* mouse models may indicate that *β*-adrenergic regulation of Ca_V_1.2 is a key point of intersection of PKA and Rad signaling pathways. This hypothesis is supported by our recent demonstration of convergent regulation of Ca_V_1.2 by PKA and Rad in transfected human cells (Hovey et al., 2022), which lends additional weight to our conclusions regarding the importance of regulation of Cav1.2 phosphorylation at physiological levels of *β*-adrenergic stimulation.

## Data Availability

The original contributions presented in the study are included in the article/supplementary material, further inquiries can be directed to the corresponding author.
