# Supplementary material for: Impairment of β-adrenergic regulation and exacerbation of pressure-induced heart failure in mice with mutations in phosphoregulatory sites in the cardiac CaV1.2 calcium channel
Source: Front Physiol. 2023 Feb 8;14:1049611. doi: 10.3389/fphys.2023.1049611 (PMC9944942; doi:10.3389/fphys.2023.1049611)
Supplement: Supplementary file 1 [file Table5.pdf]

Supplementary Table S5. Heart rate response to increasing doses of isoproterenol in mice with Cav1.2 phosphomutations. Baseline and isoproterenol-stimulated heart rate and change in heart rate. Statistical significance determined via ANOVA with Tukey post-hoc tests.

	WT	S1700A	STAA	S1928A	STAA (+/-)
0.25 µg/kg Cohort	*N = 11*	*N = 8*	*N = 6*	*N = 8*	*N = 8*
$HR_{Baseline}$ ± SEM ($BPM*10^{-2}$)	4.9 ± 0.2	5.3 ± 0.1	5.0 ± 0.4	4.1 ± 0.1	4.7 ± 0.1
$HR_{Iso, 0.25 µg/kg}$ ± SEM ($BPM*10^{-2}$)	5.2 ± 0.2	5.5 ± 0.1	5.6 ± 0.3	4.4 ± 0.2	4.7 ± 0.2
p-value HR_{Iso} vs Baseline	0.96	0.99	0.70	0.99	1.0
p-value HR_{Iso} vs WT HR_{iso}		0.80	0.66	**0.04**	0.36
p-value HR_{Iso} vs STAA HR_{iso}	0.66	1.0		**0.004**	**0.05**
ΔHR ± SEM ($BPM*10^{-2}$)	0.3 ± 0.1	0.2 ± 0.1	0.6 ± 0.3	0.3 ± 0.2	0.03 ± 0.1
p-value ΔHR vs WT		0.99	0.68	1.0	0.69
p-value ΔHR vs STAA	0.68	0.47		0.72	0.15
1 µg/kg Cohort	N = 6	N = 7	*N = 8*	*N = 6*	*N = 9*
$HR_{Baseline}$ ± SEM ($BPM*10^{-2}$)	4.4 ± 0.2	5.3 ± 0.1	5.3 ± 0.2	4.2 ± 0.2	4.6 ± 0.1
$HR_{Iso, 1 µg/kg}$ ± SEM ($BPM*10^{-2}$)	5.3 ± 0.2	6.3 ± 0.3	6.3 ± 0.2	5.1 ± 0.2	5.3 ± 0.2
p-value; HR_{Iso} vs Baseline	0.11	**0.022**	**0.011**	0.11	0.15
p-value HR_{Iso} vs WT HR_{iso}		**0.041**	**0.034**	0.98	1.0
p-value HR_{Iso} vs STAA HR_{iso}	**0.034**	1.0		**0.007**	**0.014**
ΔHR ± SEM ($BPM*10^{-2}$)	0.9 ± 0.1	1.0 ± 0.3	1.0 ± 0.3	0.8 ± 0.2	0.7 ± 0.2
p-value ΔHR vs WT		1.0	1.0	1.0	0.98
p-value ΔHR vs STAA	1.0	1.0		0.98	0.88
100 µg/kg Cohort	*N = 7*	*N = 7*	*N = 7*	*N = 7*	*N = 11*
$HR_{Baseline}$ ± SEM ($BPM*10^{-2}$)	5.0 ± 0.2	5.3 ± 0.1	4.9 ± 0.3	4.0 ± 0.1	5.1 ± 0.1
$HR_{Iso, 100 µg/kg}$ ± SEM ($BPM*10^{-2}$)	6.4 ± 0.1	6.6 ± 0.3	6.6 ± 0.2	6.0 ± 0.2	6.1 ± 0.1
p- value HR_{Iso} vs Baseline	**< 0.001**	**< 0.001**	**< 0.001**	**< 0.001**	**< 0.001**
p-value HR_{Iso} vs WT HR_{iso}		0.96	0.96	0.67	0.80
p-value HR_{Iso} vs STAA HR_{iso}	0.96	1.0		0.29	1.0
ΔHR ± SEM ($BPM*10^{-2}$)	1.3 ± 0.2	1.3 ± 0.2	1.7 ± 0.3	2.0 ± 0.2	1.0 ± 0.1
p-value ΔHR vs WT		1.0	0.65	0.13	0.78
p-value ΔHR vs STAA	0.65	0.65		0.83	0.08
