# Supplementary material for: Impairment of β-adrenergic regulation and exacerbation of pressure-induced heart failure in mice with mutations in phosphoregulatory sites in the cardiac CaV1.2 calcium channel
Source: Front Physiol. 2023 Feb 8;14:1049611. doi: 10.3389/fphys.2023.1049611 (PMC9944942; doi:10.3389/fphys.2023.1049611)
Supplement: Supplementary file 2 [file Table4.pdf]

Supplementary Table S4. Dependence of baseline cardiac parameters on sex in mice with $Ca_V1.2$ phosphoregulatory mutations. Baseline ventricular fractional shortening, heart rate, left ventricular end-diastolic diameter and end-systolic diameter in male and female WT, heterozygous and homozygous S1700A and STAA, and S1928A animals aged 30-120 days.

	**WT**	**WT**	**S1700A**	**S1700A**	**STAA**	**STAA**	**S1928A**	**S1928A**	**STAA (+/-)**	**STAA (+/-)**
Baseline	*M (n = 14)*	*F (n = 25)*	*M (n = 10)*	*F (n = 18)*	*M (n = 17)*	*F (n = 13)*	*M (n = 7)*	*F (n = 12)*	*M (n = 9)*	*F (n = 11)*
**FS ± SEM (%)**	33 ± 1	30.6 ± 0.5	19 ± 1	17 ± 1	19 ± 1	20 ± 1	26 ± 2	28 ± 1	28 ± 1	28 ± 1
p-value FS vs WT (same sex)			**< 0.001**	**< 0.001**	**< 0.001**	**< 0.001**	**0.003**	0.97	0.14	0.77
p-value M vs F	0.77	0.77	0.98	0.98	0.98	0.98	0.75	0.75	1.0	1.0
**HR ± SEM (BPM*10^{-2})**	4.7 ± 0.1	4.7 ± 0.1	5.2 ± 0.1	5.1 ± 0.1	5.0 ± 0.1	5.0 ± 0.2	4.3 ± 0.1	4.0 ± 0.1	4.5 ± 0.1	4.7 ± 0.1
p-value FS vs WT (same sex)			0.17	0.10	0.68	0.6	0.82	**0.002**	1.0	1.0
p-value M vs F	1.0	1.0	1.0	1.0	1.0	1.0	0.81	0.81	0.99	0.99
**LVEDD ± SEM (mm)**	61 ± 3	58 ± 3	75 ± 7	77 ± 6	91 ± 7	66 ± 4	81 ± 5	65 ± 5	69 ± 5	63 ± 4
p-value FS vs WT (same sex)			0.83	0.10	**0.004**	0.98	0.31	0.27	1.0	1.0
p-value M vs F	1.0	1.0	1.0	1.0	**0.04**	**0.04**	1.0	1.0	1.0	1.0
**LVESD ± SEM (mm)**	23 ± 1	24 ± 1	47 ± 5	51 ± 5	56 ± 5	40 ± 4	41 ± 5	30 ± 3	32 ± 3	29 ± 2
p-value FS vs WT (same sex)			**0.004**	**< 0.001**	**< 0.001**	**0.049**	0.091	0.38	0.91	0.99
p-value M vs F	1.0	1.0	1.0	1.0	0.09	0.09	0.99	0.99	1.0	1.0
