# Supplementary material for: Impairment of β-adrenergic regulation and exacerbation of pressure-induced heart failure in mice with mutations in phosphoregulatory sites in the cardiac CaV1.2 calcium channel
Source: Front Physiol. 2023 Feb 8;14:1049611. doi: 10.3389/fphys.2023.1049611 (PMC9944942; doi:10.3389/fphys.2023.1049611)
Supplement: Supplementary file 3 [file Table1.pdf]

Supplementary Table S1. Baseline cardiac parameters in mice with $Ca_V1.2$ phosphoregulatory site mutations. *Top*. Baseline ventricular fractional shortening, heart rate, left-ventricular end-diastolic and end-systolic diameter, and heart weight in WT, heterozygous and homozygous S1700A and STAA, and S1928A animals aged 30-120 days. *Bottom*. Baseline and isoproterenol-stimulated fractional shortening and change in fractional shortening. Statistical significance determined via ANOVA with Tukey post-hoc tests.

	**WT**	**S1700A**	**STAA**	**S1928A**	**STAA (+/-)**	**S1700A (+/-)**
Baseline	*N = 41*	*N = 28*	*N = 53*	*N = 19*	*N = 23*	*N = 21*
**FS ± SEM (%)**	31.7 ± 0.6	17.9 ± 0.8	18.3 ± 0.6	27 ± 1	27.6 ± 0.6	26.7 ± 0.9
p-value FS vs WT		**< 0.001**	**< 0.001**	**0.001**	**0.0020**	**< 0.001**
p-value FS vs STAA	**< 0.001**	1.0		**< 0.001**	**< 0.001**	**< 0.001**
**HR ± SEM (BPM)**	468 ± 7	515 ± 6	504 ± 7	410 ± 8	468 ± 9	486 ± 9
p-value HR vs WT		**< 0.001**	**0.001**	**< 0.001**	1.0	0.64
p-value HR vs STAA	**0.001**	0.88		**< 0.001**	0.014	0.60
**LVEDD ± SEM (mm)**	3.70 ± 0.05	4.1 ± 0.1	4.22 ± 0.06	4.00 ± 0.09	3.96 ± 0.09	3.68 ± 0.08
p-value LVEDD vs WT		0.002	**< 0.001**	0.11	0.16	0.99
p-value LVEDD vs STAA	**< 0.001**	0.82		0.36	0.13	**< 0.001**
**LVESD ± SEM (mm)**	2.52 ± 0.04	3.3 ± 0.1	3.44 ± 0.07	2.9 ± 0.1	2.88 ± 0.08	2.67 ± 0.09
p-value LVESD vs WT		**< 0.001**	**< 0.001**	**0.023**	**0.021**	0.79
p-value LVESD vs STAA	**< 0.001**	0.74		**< 0.001**	**< 0.001**	**< 0.001**
Baseline	*N = 10*	*N = 14*	*N =15*	*N = 19*	*N = 18*	*N = 8*
**HW/BW ± SEM (mg/g)**	4.7 ± 0.2	6.2 ± 0.1	6.6 ± 0.2	5.8 ± 0.4	5.3 ± 0.3	5.3 ± 0.3
p-value HW/BW vs WT		0.054	**0.005**	0.23	0.83	0.91
p-value HW/BW vs STAA	**0.005**	0.96		0.44	**0.04**	0.18
0.25 μg/kg Cohort	*N = 11*	*N = 8*	*N = 6*	*N = 8*	*N = 8*	
**$FS_{Baseline}$ ± SEM (%)**	31.2 ± 0.8	20 ± 2	20 ± 2	26 ± 2	26 ± 1	
**$FS_{Iso,\ 0.25\ \mu g/kg}$ ± SEM (%)**	45 ± 3	26 ± 3	25 ± 1	32.1 ± 3	32 ± 1	
p-value FS_{Iso} vs Baseline	**< 0.001**	0.69	0.98	**0.040**	0.76	
p-value FS_{Iso} vs WT FSi_{so}		**< 0.001**	**< 0.001**	**0.015**	**0.008**	
**ΔFS ± SEM (%)**	14 ± 2	6 ± 1	4 ± 1	6 ± 3	6 ± 1	
p-value ΔFS vs WT		**0.027**	**0.008**	**0.027**	**0.027**	
p-value ΔFS vs STAA	**0.008**	0.96		0.96	0.96	
1 μg/kg Cohort	*N = 6*	*N = 7*	*N = 8*	*N = 6*	*N = 9*	
**$FS_{Baseline}$ ± SEM (%)**	31 ± 1	19 ± 2	19 ± 2	29 ± 2	28 ± 1	
**$FS_{Iso,\ 1\ \mu g/kg}$ ± SEM (%)**	51 ± 3	35 ± 4	34 ± 4	48 ± 4	46 ± 2	
p-value; FS_{Iso} vs Baseline	**< 0.001**	**0.039**	**0.007**	**0.002**	**< 0.001**	
p-value FS_{Iso} vs WT FSi_{so}		**0.021**	**0.008**	0.97	0.83	
**ΔFS ± SEM (%)**	20 ± 3	16 ± 3	15 ± 3	18 ± 4	18 ± 2	
p-value ΔFS vs WT		0.89	0.76	0.99	0.99	
p-value ΔFS vs STAA		1.00		0.95	0.93	
100 μg/kg Cohort	*N = 7*	*N = 7*	*N = 7*	*N = 7*	*N = 11*	
**$FS_{Baseline}$ ± SEM (%)**	33 ± 1	19 ± 2	19 ± 2	25 ± 1	26.3 ± 0.9	
**$FS_{Iso,\ 100\ \mu g/kg}$ ± SEM (%)**	60 ± 4	51 ± 3	45 ± 3	53 ± 6	57 ± 3	

p-value FS_{Iso} vs Baseline	**< 0.001**	**< 0.001**	**< 0.001**	**< 0.001**	**< 0.001**
p-value FS_{Iso} vs WT FSi_{so}		0.61	0.12	0.82	1
**ΔFS ± SEM (%)**	26 ± 3	32 ± 4	26 ± 3	28 ± 5	31 ± 2
p-value ΔFS vs WT		0.75	1.0	0.99	0.80
p-value ΔFS vs STAA		0.75		0.99	0.80
