# Supplementary material for: Impairment of β-adrenergic regulation and exacerbation of pressure-induced heart failure in mice with mutations in phosphoregulatory sites in the cardiac CaV1.2 calcium channel
Source: Front Physiol. 2023 Feb 8;14:1049611. doi: 10.3389/fphys.2023.1049611 (PMC9944942; doi:10.3389/fphys.2023.1049611)
Supplement: Supplementary file 4 [file Table2.pdf]

Supplementary Table S2. ***In vivo*** **contractile response to isoproterenol in mice with $Ca_V1.2$ phosphoregulatory site mutations.** *Top*. Baseline and post-TAC ventricular fractional shortening, change in fractional shortening, and left-ventricular end-diastolic and end-systolic diameter, post-TAC heart weight. *Bottom*. Expression fold-change (R_Q) in hypertrophic markers ANP, BNP, and β-MHC ventricular tissue from post-TAC mice relative to age-matched mice that did not receive surgery. Statistical significance determined via ANOVA with Tukey post-hoc tests.

	**WT**	**STAA**	**S1700A**	**STAA (+/-)**	**S1700A (+/-)**
	N = 6	*N = 5*	*N = 5*	*N = 7*	*N = 5*
**$FS_{Baseline}$ ± SEM (%)**	30.1 ± 0.8	23.9 ± 2	16.9 ± 0.9	28.8 ± 0.9	24 ± 1
**FS_{4W} ± SEM (%)**	18 ± 2	9 ± 2		21 ± 1	16 ± 1
p-value FS_{4W} vs $FS_{Baseline}$	**< 0.001**	**< 0.001**		**0.013**	**0.016**
**ΔFS ± SEM (%)**	-13 ± 1	-15 ± 2		-8 ± 1	-7 ± 2
p-value ΔFS vs WT		0.78		0.080	**0.048**
p-value ΔFS vs STAA	0.78			**0.014**	**0.009**
**$LVEDD_{Baseline}$ ± SEM (mm)**	3.7 ± 0.1	4.07 ± 0.09	4.3 ± 0.1	3.9 ± 0.1	3.92 ± 0.03
**$LVEDD_{4W}$ ± SEM (mm)**	4.2 ± 0.1	5.2 ± 0.2		4.0 ± 0.1	4.5 ± 0.2
p-value $LVEDD_{4W}$ vs $LVEDD_{Baseline}$	0.081	< 0.001		0.99	0.093
**HW/BW_{4W} ± SEM (mg/g)**	5.9 ± 0.5	8.4 ± 0.3		5.6 ± 0.5	6.3 ± 0.9
p-value HW/BW_{4W} vs No TAC	0.42	**0.047**		1.0	0.79
# Animals Receiving TAC	N = 10	N = 10	N = 7	N = 11	N = 10
# Animals Alive 4 Weeks Post-TAC	N = 6	N = 5	N = 0	N = 7	N = 5
<u>*ANP*</u>					
**$\Delta C_{T, No TAC}$ ± SEM**	6.5 ± 0.6; N = 13	5.8 ± 0.8; N = 14		3.2 ± 0.3; N = 9	3.3 ± 0.4; N = 9
**$\Delta C_{T, TAC}$ ± SEM**	5.3 ± 0.4; N = 5	3.0 ± 0.4; N = 5		3.0 ± 0.7; N = 5	3.2 ± 0.7; N = 5
**R_Q [$2^{-(\Delta CT,TAC - \Delta CT,NoTAC)}$] ± SEM**	2.7 ± 0.4	8 ± 2		2.2 ± 0.9	1.7 ± 0.7
p-value R_Q vs WT		**0.039**		1.0	0.90
<u>*BNP*</u>					
**$\Delta C_{T, No TAC}$ ± SEM**	6.3 ± 0.4 (N = 14)	5.2 ± 0.5 (N = 14)		4.8 ± 0.3 (N = 9)	5.4 ± 0.3 (N = 9)
**$\Delta C_{T, TAC}$ ± SEM**	6.2 ± 0.34 (N = 5)	4.8 ± 0.5 (N = 5)		5.5 ± 0.4 (N = 5)	5.1 ± 0.5 (N = 5)
**R_Q [$2^{-(\Delta CT,TAC - \Delta CT,NoTAC)}$] ± SEM**	1.2 ± 0.3	1.8 ± 0.8		0.7 ± 0.2	1.5 ± 0.4
p-value R_Q vs WT		0.78		0.86	0.90
<u>*β-MHC*</u>					
**$\Delta C_{T, No TAC}$ ± SEM (*N*)**	7.0 ± 0.5 (N = 13)	5.9 ± 0.4 (N = 14)		7.6 ± 0.4 (N = 8)	7.2 ± 0.6 (N = 9)
**$\Delta C_{T, TAC}$ ± SEM (*N*)**	6.6 ± 0.4 (N = 5)	4.9 ± 0.5 (N = 5)		6.9 ± 0.6 (N = 5)	6.2 ± 0.1 (N = 5)
**R_Q [$2^{-(\Delta CT,TAC - \Delta CT,NoTAC)}$] ± SEM**	1.6 ± 0.4	3 ± 1		2.2 ± 0.8	2.1 ± 0.2
p-value R_Q vs WT		0.66		1.0	0.90
