# Supplementary material for: Impairment of β-adrenergic regulation and exacerbation of pressure-induced heart failure in mice with mutations in phosphoregulatory sites in the cardiac CaV1.2 calcium channel
Source: Front Physiol. 2023 Feb 8;14:1049611. doi: 10.3389/fphys.2023.1049611 (PMC9944942; doi:10.3389/fphys.2023.1049611)
Supplement: Supplementary file 5 [file Image2.pdf]

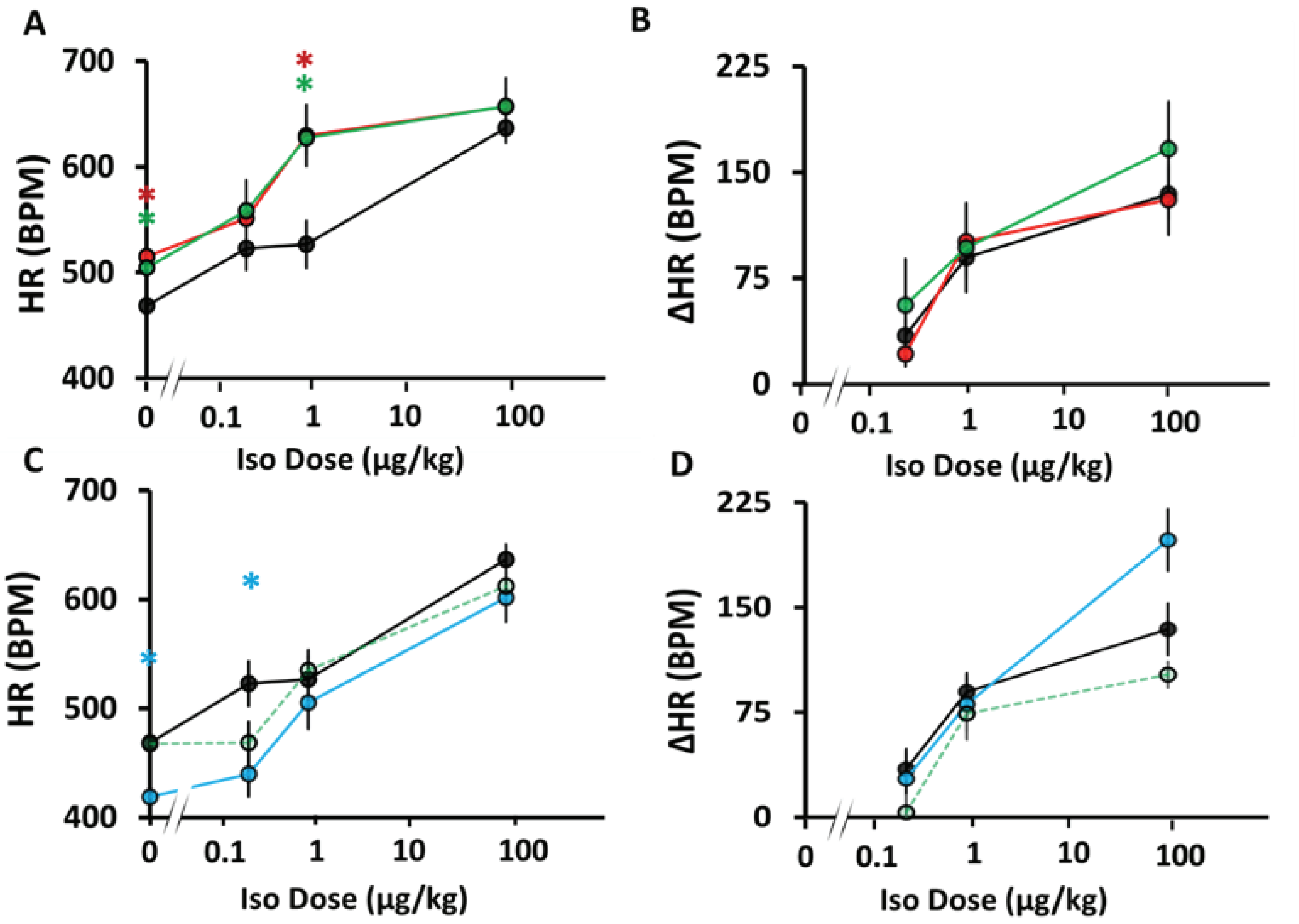

Supplementary Figure S2. Heart rate response to increasing doses of isoproterenol in mice with $Ca_V1.2$ phosphoregulatory mutations. (A) Isoproterenol dose-response assessed by heart rate in WT (black), STAA (green), and S1700A (red) mice. (B) Isoproterenol dose-response assessed by change in heart rate in WT, STAA, and S1700A mice. (C) Isoproterenol dose-response assessed by heart rate in WT (black), $STAA^{+/-}$ (light green), and S1928A (light blue) mice. (D) Isoproterenol dose-response assessed by change in heart rate in WT, $STAA^{+/-}$, and S1928A mice. Isoproterenol was administered via intraperitoneal injection of the indicated doses. Statistical significance was determined via ANOVA with Tukey post-hoc tests. Error bars are S.E.M.; 0.25 μg/kg, N = 11, 8, 6, 8, 8 (for WT, S1700A, STAA, S1928A, $STAA^{+/-,}$ respectively); 1 μg/kg, N = 6, 7, 8, 6, 9 (for WT, S1700A, STAA, S1928A, $STAA^{+/-}$, respectively); 100 μ g/kg, N = 7, 7, 7, 7, 11 (for WT, S1700A, STAA, S1928A, $STAA^{+/-}$, respectively); * $p< 0.05$ (red * S1700A vs WT, light-green STAA +/- vs WT, dark green STAA vs WT, light-blue S1928A vs WT).
