# Supplementary material for: Impairment of β-adrenergic regulation and exacerbation of pressure-induced heart failure in mice with mutations in phosphoregulatory sites in the cardiac CaV1.2 calcium channel
Source: Front Physiol. 2023 Feb 8;14:1049611. doi: 10.3389/fphys.2023.1049611 (PMC9944942; doi:10.3389/fphys.2023.1049611)
Supplement: Supplementary file 6 [file Table3.pdf]

Supplementary Table S3. Additional baseline echocardiographic parameters in mice with $Ca_V1.2$ phosphoregulatory mutations. Interventricular septal end diastole (IVSd) and interventricular septal end systole (IVSs), left ventricular posterior wall end diastole (LVPWd) and left ventricular posterior end systole (LVPWs), and left ventricular mass. WT, heterozygous and homozygous S1700A and STAA, and S1928A animals aged 30-120 days.

	**WT**	**S1700A**	**STAA**	**S1928A**	**STAA (+/-)**	**S1700A (+/-)**
Baseline	*N = 41*	*N = 28*	*N = 53*	*N = 19*	*N = 23*	*N = 21*
**IVS_d**	0.62 ± 0.02	0.63 ± 0.03	0.67 ± 0.02	0.68 ± 0.04	0.64 ± 0.03	0.64 ± 0.04
p-value vs WT		1.0	0.61	0.71	1.0	1.0
p-value vs STAA	0.61	0.87		1.0	0.97	0.96
**IVS_s**	0.91 ± 0.02	0.79 ± 0.03	0.86 ± 0.02	0.93 ± 0.04	0.87 ± 0.03	0.87 ± 0.04
p-value vs WT		**0.018**	0.61	1.0	0.91	0.92
p-value vs STAA	0.61	0.36		0.52	1.0	1.0
**$LVPW_d$**	0.61 ± 0.02	0.64 ± 0.03	0.66 ± 0.02	0.60 ± 0.02	0.62 ± 0.04	0.60 ± 0.03
p-value vs WT		0.96	0.56	1.0	1.0	1.0
p-value vs STAA	0.56	0.99		0.63	0.88	0.60
**$LVPW_s$**	0.75 ± 0.02	0.70 ± 0.02	0.74 ± 0.02	0.71 ± 0.03	0.78 ± 0.03	0.73 ± 0.03
p-value vs WT		0.65	0.99	0.89	0.96	0.99
p-value vs STAA	0.99	0.80		0.96	0.84	1.0
**LV Mass**	75 ± 4	98 ± 7	102 ± 9	97 ± 8	81 ± 8	76 ± 6
p-value vs WT		0.29	**0.046**	0.48	1.0	1.0
p-value vs STAA	**0.046**	1.0		1.0	0.41	0.22
