# Supplementary material for: Impairment of β-adrenergic regulation and exacerbation of pressure-induced heart failure in mice with mutations in phosphoregulatory sites in the cardiac CaV1.2 calcium channel
Source: Front Physiol. 2023 Feb 8;14:1049611. doi: 10.3389/fphys.2023.1049611 (PMC9944942; doi:10.3389/fphys.2023.1049611)
Supplement: Supplementary file 7 [file Image1.pdf]

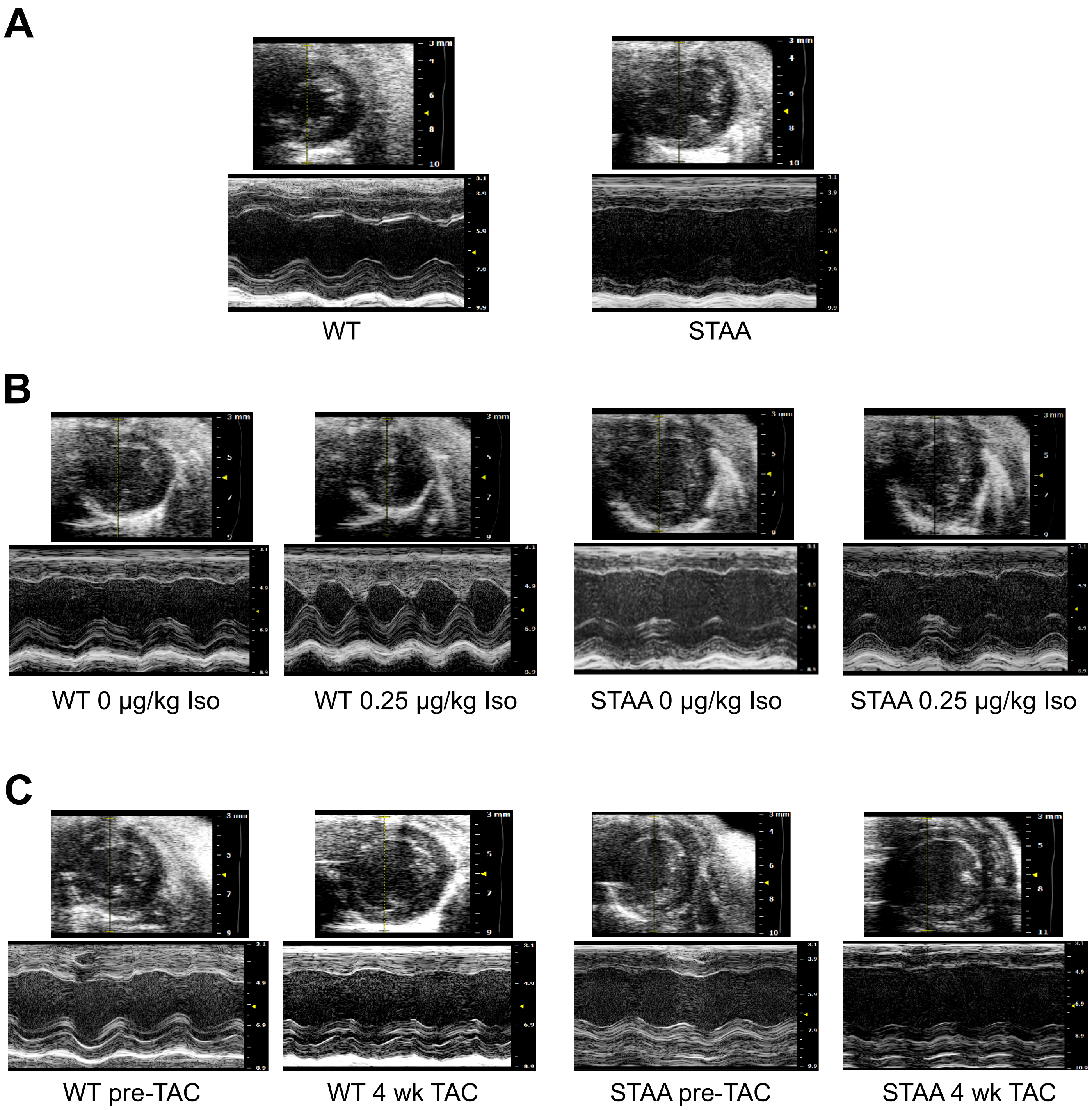

Supplemental Figure S1. Representative echocardiographic M-mode images. (A) WT and STAA mice at baseline. (B) WT and STAA mice subjected to Iso administration. (C) WT and STAA mice subjected to 4 weeks of TAC.
